# Unveiling socio-demographic determinants of low birth weight using machine learning techniques

**DOI:** 10.1371/journal.pgph.0005745

**Published:** 2026-01-07

**Authors:** Mohammad Safi Uddin, Md. Refath Islam, K. M. Ariful Kabir

**Affiliations:** 1 Directorate General of Family Planning, Ministry of Health and Family Welfare, Dhaka, Bangladesh,; 2 Department of Mathematics, Bangladesh University of Engineering and Technology, Dhaka, Bangladesh; Yale University, UNITED STATES OF AMERICA

## Abstract

Low birth weight (LBW) poses significant challenges to child survival, contributing to increased rates of mortality and morbidity, and has long-term adverse effects on overall health. The persistently high prevalence of LBW in low- and middle-income countries, including Bangladesh, reflects underlying health disparities. Despite recent improvements, Bangladesh still reports a notable LBW rate of 14.5%, indicating persistent maternal and child health concerns. Various socio-demographic factors influence birth weight, necessitating a comprehensive investigation into their contributions. This study aims to identify the key determinants of LBW and develop a machine learning-based predictive model to assess vulnerable mothers of having LBW babies based on risk factors associated with birth weight. Data for this study were obtained from the Bangladesh Demographic and Health Survey (BDHS) 2022, which encompassed 2,621 women (excluding missing cases) and 8,784 women (including missing cases). Several machine learning algorithms, including logistic regression, Naïve Bayes, k-nearest neighbors (KNN), random forest, support vector machine (SVM), Lasso regression, regression tree, neural networks, XGBoost, AdaBoost, and decision tree classifiers, were employed to analyze the risk factors. Model performance was evaluated using a train-test split approach and 10-fold cross-validation, with accuracy, precision, recall, F1-score, R² score (only for the regression model), and mean squared error (MSE) as assessment metrics. The findings indicate that ‘Age at first birth’ and ‘Education Level’ emerged as the most influential predictors of LBW, while AdaBoost demonstrated the highest predictive accuracy among the applied models. The findings of this study might make significant contributions in identifying vulnerable mothers giving birth to children with LBW and making policies highlighting risk factors responsible for LBW to reduce the frequency of LBW.

## Introduction

Low birth weight (LBW) is a critical outcome indicator recognized in the Global Nutrition Monitoring Framework and WHO’s core health indicators, playing a pivotal role in advancing Sustainable Development Goals related to reproductive health [1–3]. In 2020, approximately 20 million newborns, accounting for 14.7% of all births that year, were born with LBW, compared to 16.6% in 2010 [4,5]. In the world, nearly one in seven newborns is affected by LBW; the prevalence is far higher than the desired target [5,6]. In 2012, the Global Nutrition Target aimed to reduce the 30% prevalence of LBW from 15% to 10.5% by 2025, which was later extended to 2030 by UNICEF and WHO [4,7]. However, progress has been slow, with a worldwide annual average reduction rate of LBW babies of only 0.30% from 2012 to 2020, far below the required rate of 1.96% per year to meet the target, and it needs to increase 11-fold to 3.30% to meet the target by 2030 [6]. Therefore, the reduction of the frequency of LBW remains a significant public health challenge globally, especially in low- and middle-income countries like Bangladesh.

South Asia recorded the most significant reduction in LBW, with prevalence declining by 4.5 percentage points over two decades, from 29.4% in 2000 to 24.9% in 2020 [8]. Despite this progress, the annual average reduction rate in low birth weight in this region from 2012 to 2020 was only 0.85%, still below the target rate [8]. Notably, over 40% of the 19.8 million babies born with LBW globally were from South Asia, highlighting the persistent inequalities in healthcare systems across regions [4,9]. Although the prevalence of LBW is declining, the rate of decrease is much slower compared to most developed and developing countries, despite the interventions implemented to reduce LBW [10].

According to the World Health Organization (WHO), LBW refers to a newborn weighing less than 2,500 grams (5.5 pounds). LBW poses both short and long-term ramifications on maternal and child health, like child mortality and other diseases of future life [11,12]. These infants faced a higher likelihood of dying within their first month, and those who survived were at increased risk of lifelong issues such as stunted growth [13], lower IQ [14], and motor delays and other behavioral problems [15], and were more likely to develop chronic non-communicable diseases in adult life [16]. Additionally, studies have revealed the detrimental effects of LBW on children, both physically and cognitively, ranging from increased risk of death to lifelong disabilities [17,18]. As a signatory to the Sustainable Development Goals (SDGs), Bangladesh aims to reduce the neonatal mortality rate (NMR) to 12 per 1,000 live births by 2030, currently at 20 per 1,000 live births [10,19]. Approximately 5.5% of infants with LBW died, compared to 2% of infants with a birth weight greater than 2500 grams [20].

Various sociodemographic (Non-health) and health-related factors to mothers, such as age, age at first birth, education, wealth level, place of residence, gestational period, occupation, number of children, and BMI, have been shown to contribute to the likelihood of having LBW babies in different settings worldwide [21–24]. As a result, it is crucial to thoroughly investigate the potential socio-demographic factors contributing to LBW in Bangladesh, and based on these factors, a predictive model can be developed to identify future vulnerable mothers who are at risk of giving birth to LBW babies.

In recent years, social research has increasingly used machine learning (ML) to move beyond traditional binary outcome models by providing flexible, data-driven approaches for classification and prediction. Unlike conventional logistic regression, ML algorithms can capture complex non-linearities and interactions among features, often resulting in higher predictive accuracy. Previous studies in Bangladesh have applied ML models to health-related classifications, such as predicting maternal BMI categories using algorithms like Logistic Regression, Decision Tree, Random Forest, and AdaBoost, with varying accuracies ranging from 85% to 88% [18,25,26]. Beyond LBW, ML has been employed to forecast pregnancy complications such as pre-eclampsia, gestational diabetes, and preterm birth [27], as well as to classify maternal risk levels using national mortality data with high discriminative performance [28]. Collectively, these studies underscore that ML offers richer predictive nuance than traditional approaches, supporting early detection, risk stratification, and data-driven decision support across maternal and fetal health domains—thereby reinforcing its relevance for LBW prediction in the Bangladeshi context. However, these studies often relied on aggregated data and lacked critical evaluation of model suitability and interpretability in policy contexts.

To address these gaps, this study investigates the socioeconomic, health, and demographic determinants of women giving birth to low birth weight (LBW) babies. It applies multiple ML algorithms including Logistic Regression, Naive Bayes, K-Nearest Neighbors (KNN), Random Forest, Support Vector Machine (SVM), Lasso Regression, Regression Tree, Neural Networks (MLP), XGBoost, AdaBoost, and Decision Tree to identify the most effective model. Model performances are evaluated using accuracy, precision, recall, and F1-score, considering interpretability and generalizability given the district-level data’s limited size and representativeness. Unlike most LBW studies on national or divisional scales, this research introduces a district-level spatial analysis that uncovers substantial within-country heterogeneity. Identifying local LBW hotspots allows for context-specific understanding of socioeconomic, environmental, and healthcare disparities. This district-oriented framework strengthens the policy relevance of ML-based insights, supporting geographically targeted maternal and child health interventions that can be scaled for other low- and middle-income countries.

## Literature review

The respondents’ demographic and socioeconomic features were found to be influential in the size of the children at birth. Delaying maternal age at first birth from teenage to adulthood may improve the size of the child and reduce perinatal morbidity and mortality, while smaller babies might be born to younger mothers, which can cause negative health outcomes in the future [29,30]. Additionally, other factors such as wealth level, education, BMI, and place of residence can also impact child health outcomes. Maternal education is closely associated with improved living conditions, effective pregnancy management, and better access to healthcare facilities, which in turn leads to better child health outcomes at birth [31]. Furthermore, individuals from higher household incomes tend to have babies with larger head sizes compared to those from the lowest household income [32].

During pregnancy, male infants tend to gain weight slightly faster than female infants, resulting in male newborns being generally larger [33]. According to the World Health Organization (WHO), the average birth weight of a full-term male baby is 7 pounds 6 ounces (3.3 kg), while the average birth weight of a full-term female is 7 pounds 2 ounces (3.2 kg) [34]. The average birth weight of a baby at 37–40 weeks ranges from 5 pounds 8 ounces to 8 pounds 13 ounces (2.5 to 4 kg). While low birth weight (LBW) does not necessarily indicate critical physical or mental development issues, it is a significant concern. LBW infants are at 20 times higher risk of mortality compared to those with normal birth weight and may face long-term health challenges, including brain injury, chronic diseases, intellectual impairment, and developmental disorders.

In Bangladesh, the prevalence of LBW was found to be 16.3%, where mothers with no formal education and those who belong to the poorest wealth quintile are more likely to have LBW children [35–38].

This study employed birth weight as the dependent variable. Children with birth weight below 2500 grams were considered low birth weight (LBW), whereas those with 2500 grams or above were regarded as average birth weight. It is common for all newborns with varied sociodemographic and maternal health conditions. To understand the factors affecting LBW, the outcome variable was categorized into a dichotomous variable, where ‘0’ represents LBW (<2500 grams) and ‘1’ represents normal birth weight (≥2500 grams). After reviewing the previous literature, this study aimed to examine the influence of ten sociodemographic features of the mother on birth weight, which were extracted from the BDHS-2022 dataset.

## Methodology

### Source of the dataset

Data were extracted from the Bangladesh Demographic and Health Survey (BDHS)-2022. The BDHS-2022 is a large-scale, nationally representative sample survey conducted across all Divisions and Districts under the stewardship of the Ministry of Health and Family Welfare (MoHFW), Government of Bangladesh, and the United States Agency for International Development (USAID). The data collection was completed for 30,330 households and 30,078 women aged 15–49 years, resulting in a response rate of 99% for the total sample. The primary objective of this survey was to provide updated and reliable information on fertility, family planning, maternal and child healthcare, childhood immunization, infant and child mortality, nutrition, HIV/AIDS-related knowledge and attitudes, women’s empowerment, and domestic violence against women. The information collected through the 2022 BDHS is intended to assist policymakers and program managers in designing and evaluating programs and strategies to improve the health of Bangladesh’s population. The survey also provides estimates for 15 major indicators relevant to the 4^th^ Health, Population and Nutrition Sector Program (4^th^ HPNSP) 2017–2022 of the MoHFW and to the Sustainable Development Goals (SDGs) for Bangladesh.

### Participants

The sampling frame used for the 2022 BDHS is the Integrated Multi-Purpose Sampling Master Sample, which was selected from a comprehensive list of enumeration areas (EAs) covering the entire country. It was prepared by the Bangladesh Bureau of Statistics (BBS) for the 2011 population census of the People’s Republic of Bangladesh. A stratified two-stage sampling technique was employed for data collection. In the first stage, 675 EAs were selected using probability proportional to the EA size. In the second stage, 45 households from each EA were selected to provide statistically reliable estimates of key demographic and health variables for both urban and rural areas, as well as for each of the eight divisions in Bangladesh. Ever-married women aged 15–49 were considered eligible for individual interviews by a group of previously trained personnel. A detailed description of the sampling design and survey procedure is provided in the final report of BDHS-2022.

### Data preparation

#### (i) Outcome variable.

Low birth weight (LBW), defined as infant weight <2,500 g at birth, is the outcome variable of this study. It was derived from the “Size of children” variable in the BDHS dataset, which captures information on the most recent births in Bangladesh within five years prior to the survey. LBW remains a major public health concern in Bangladesh, contributing substantially to neonatal morbidity and mortality. Identifying women at high risk of delivering LBW infants, therefore, holds strong preventive and policy value.

**Fig 1:**
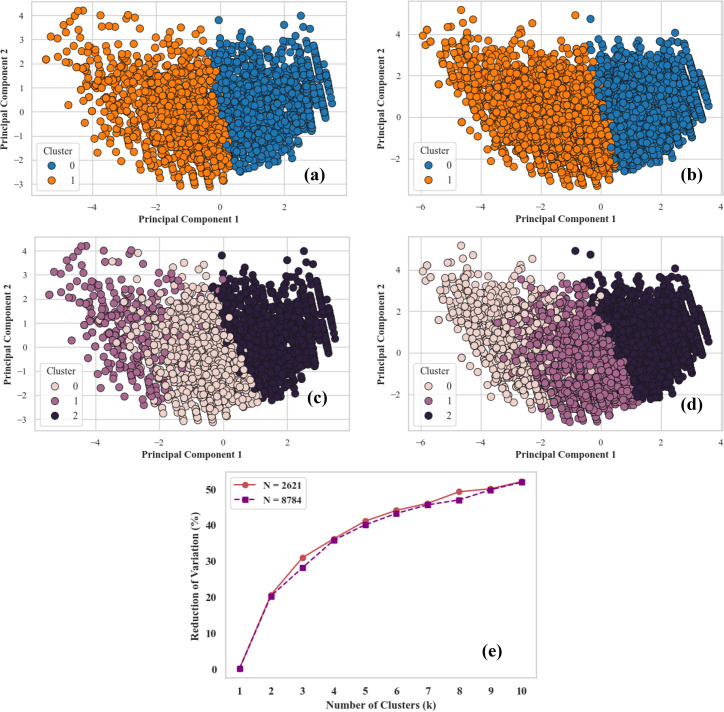
Analyzing Clusters using PCA and K-Means on Datasets of Varying Sizes. The clustering findings for a dataset size of N = 2621 are shown in subfigures (a) and (c). Clustering results are shown in subfigures (b) and (d) for dataset size N = 8784. Projected onto the first two principal components, K-Means clustering with k = 2 is shown in (a) and (b), while K-Means clustering with k = 3 is shown in (c) and (d). The decrease of variation (%) for varying numbers of clusters, k, comparing the two dataset sizes, is displayed in subfigure (e). (BDHS-2022).

In the original BDHS dataset, the variable *“Size of children”* included five categories: *very large*, *larger than average*, *average*, *smaller than average*, and *minimal*. For analysis, these were first grouped into three classes—*smaller than average*, *average*, and *larger than average*—to facilitate clustering and elbow analysis (Fig 1). Finally, they were reclassified into two groups for binary classification: *0 = low birth weight (LBW)* and *1 = normal birth weight (NBW)*.

Principal Component Analysis (PCA) was applied to assess the suitability of these categorizations [39] by reducing data complexity and identifying patterns that best distinguish between birth weight categories. The binary classification produced clearer and more distinct clusters than the three-category model, indicating stronger interpretability and better variance explanation. Therefore, the dichotomous representation (0 = LBW, 1 = NBW) was adopted for all machine learning analyses to ensure simplicity and analytical robustness.

#### (ii) Predictor variables.

The BDHS-2022 dataset initially included a comprehensive array of demographic, socioeconomic, health, and nutritional characteristics for each respondent. To identify the most relevant predictors of low birth weight (LBW), a combination of literature-based justification and empirical evaluation was employed. Drawing on previous studies on maternal and child health outcomes [27–36] and supported by preliminary statistical analyses [25,26], ten variables were selected as key determinants of LBW. These include the respondent’s age, type of place of residence, education level, wealth index, internet use, number of children ever born, age at first birth, body mass index (BMI), husband’s education level, and literacy (Table 1). Several variables were reclassified to improve analytical clarity. Respondents were first divided into five wealth categories—*poorest*, *poorer*, *middle*, *richer*, and *richest*—and then merged into three broader groups: *poor*, *middle*, and *rich*. Two continuous variables were also discretized for interpretability: *number of children ever born* (≤ 2 vs. > 2) and *age at first birth* (teenage ≤ 19 years vs. adult > 19 years). The *age of respondents* was categorized into five-year intervals: ≤ 19, 20–24, 25–29, 30–34, and ≥ 35 years. The Body Mass Index (BMI) was converted into three categories: underweight (<18.5 kg/m²), normal (18.5 kg/m²), and overweight (>18.5 kg/m²). All remaining variables were retained in their original form to preserve data integrity.

**Table 1:** The name of the features with their attributes (BDHS-2022).

Attribute Name	Name of feature	Value Type	Feature Type
M18	Size of children	Ordinal	Dependent/ Target
V012	Age of respondent	Nominal	Independent
V025	Type of place of residence	Nominal categorical	Independent
V106	Respondent’s education	Nominal categorical	Independent
V190	Wealth index	Nominal categorical	Independent
V171A	Internet use	Nominal categorical	Independent
V201	Number of children ever born	Nominal	Independent
V212	Age at first birth	Nominal	Independent
V445	Body Mass Index (BMI)	Nominal categorical	Independent
S904A	Education level of husband	Nominal categorical	Independent
V155	Literacy	Nominal categorical	Independent

### Sample size

The BDHS-2022 dataset included 8,784 women of reproductive age. Because two key variables—*Body Mass Index (BMI)* and *birth weight*—contained missing entries, two analytical subsets were created: one complete-case dataset (N = 2,621) and one full dataset (N = 8,784)

### Missing data

The BDHS-2022 dataset exhibited a notable degree of missingness across several key variables, posing an inherent limitation to the analysis. Among the 8,784 respondents, information on child size at birth was unavailable for 3,442 cases, while Body Mass Index (BMI) data was missing for 4,304 respondents accounting for approximately 39% of the total sample. Additionally, husband’s education level showed around 2% missing values. To address these gaps without discarding valuable observations, mode imputation was employed, given that most variables were categorical in nature. This approach replaces missing entries with the most frequently occurring category, preserving the dataset’s overall structure and maximizing statistical power by maintaining the full sample size. However, while mode imputation effectively mitigates data loss, it can marginally underestimate natural variability and introduce subtle bias by overrepresenting dominant categories. Consequently, the imputed dataset should be interpreted with caution, acknowledging that although this method enhances completeness and model stability, it may slightly distort underlying relationships within the data.

**Fig 2:**
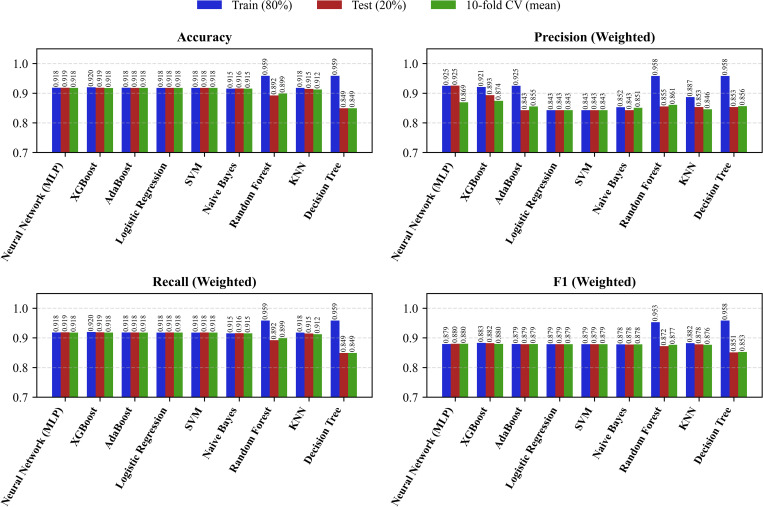
Performance comparison of eight machine learning classifiers after mode imputation (N = 8,784). Accuracy, Precision, Recall, and F1-score are shown for training (80%), test (20%), and 10-fold cross-validation (CV) datasets. The figure illustrates the consistency and generalization of model performance across different evaluation schemes following the imputation process.

Maternal body mass index (BMI) had significant missing data, with around 39% unrecorded entries. We created two analytical datasets to assess how this affected our models: a complete-case dataset with 2,621 observations, ensuring internal validity by including only cases with complete information, and a full dataset with all 8,784 observations, enhancing generalizability by including those with missing BMI values. We conducted comparative analyses between these datasets to evaluate how different approaches to handling missing data influenced model outcomes and the sensitivity of our findings. Fig 2 demonstrates the influence of mode imputation on model performance across the training, single test, and 10-fold cross-validation (CV) datasets, evaluated using Accuracy, Precision, Recall, and F1-score (N = 8,784). Although mode imputation enables the retention of all available observations, thereby avoiding the data loss inherent in complete-case analysis, it introduces a subtle yet systematic bias by disproportionately reinforcing the dominant category. This bias can distort the underlying feature distributions and reduce variability, especially when missingness is not entirely random. As a result, flexible models such as Decision Tree, Random Forest, and K-Nearest Neighbors tend to overfit the imputed data performing exceptionally well on training sets but showing noticeable performance drops in testing and CV evaluations. This divergence reflects their sensitivity to artificial regularities introduced by imputation.

In contrast, more robust learners like Logistic Regression, Support Vector Machine, Multilayer Perceptron, AdaBoost, and XGBoost maintain consistent performance across datasets, suggesting better resilience to the noise and distributional shifts caused by mode imputation. Among the evaluated metrics, Precision exhibited the most significant fluctuation, indicating that imputation can artificially sharpen class boundaries and misrepresent the true separability of outcomes. It is critical to balance data completeness and fidelity to minimize bias. While complete-case analysis avoids imputation bias by relying solely on observed data, it can reduce statistical power and introduce selection bias if missingness is systematically related to key variables. Conversely, mode imputation maintains sample size but risks distorting the data-generating process. Therefore, a hybrid approach such as comparing results from both methods, conducting sensitivity analyses, or employing more sophisticated imputation techniques (e.g., multiple imputation or model-based imputation) can help ensure that model estimates and interpretations remain unbiased and generalized.

### Model development and validation

For model evaluation, the dataset was partitioned using two complementary approaches to ensure the robustness and reliability of the results. Initially, an 80/20 train–test split was employed, where 80% of the data were used for model training and 20% were reserved exclusively for testing the model’s predictive performance on unseen data [25]. This approach enabled an unbiased assessment of model generalization and helped identify potential overfitting or underfitting tendencies during the development phase. A 10-fold cross-validation scheme to ensure robustness and generalizability of the results. Hyperparameter tuning was performed through repeated 10-fold cross-validation with grid search within the training data to determine optimal parameters and minimize overfitting. To develop a reliable and interpretable prediction framework, a range of machine learning (ML) algorithms were compared, including Logistic Regression (LR), Naive Bayes (NB), K-Nearest Neighbors (KNN), Random Forest (RF), Support Vector Machine (SVM), Lasso Regression (LaR), Regression Tree (RT), Neural Network (MLP), XGBoost, AdaBoost, and Decision Tree (DT). R² and MSE metrics were computed solely for the two regression-based models—Lasso Regression and Regression Tree—to assess their predictive fit. In contrast, classification models were evaluated using accuracy, precision, recall, and F1-score.

Machine learning (ML) has increasingly been applied in maternal health research to predict outcomes such as low birth weight (LBW), maternal anemia, and neonatal mortality, offering improved accuracy and interpretability compared to traditional methods. Studies employing Logistic Regression and Decision Trees have effectively modeled categorical and binary health outcomes [18,39]. At the same time, ensemble methods such as Random Forest and XGBoost have demonstrated superior predictive power and robustness in managing high-dimensional and imbalanced data [26,40]. Building on this literature, the present study selected algorithms based on their established strengths and relevance to maternal health prediction: Logistic Regression for interpretability, Naive Bayes for efficiency with categorical data, K-Nearest Neighbors for capturing local nonlinearities, Lasso Regression for feature selection and model stability [6], Decision Trees for rule-based interpretability, and Multilayer Perceptron for modeling complex interactions. Ensemble models Random Forest, XGBoost, and AdaBoost were further employed to enhance accuracy, with AdaBoost adapting to difficult-to-classify cases. Collectively, this approach integrates diverse ML paradigms to identify key determinants of LBW while strengthening the methodological rigor and policy relevance of district-level maternal health analysis.

One statistical approach for binary classification is logistic regression. It simulates the likelihood of a specific result. Y∈{0,1} utilizing the sigmoid function as a function of the input characteristics X.


$$P\left(Y=\text{1}|X\right)=\frac{\text{1}}{\text{1}\;+\;e^{-(\beta_{\text{0}}+\;\beta^{T}X)}}\;\;\;\;\;\;\;\;\;\;\;$$ (1.1)

Here, $\beta_{\text{0}}$ is the interception, β is the vector of coefficients, and X is the feature vector. The log-likelihood function is maximized while estimating the parameters β using Maximum Likelihood Estimation (MLE):


$$\begin{array}{c} l(\beta )=\;\sum_{i=\text{1}}^{n}\left[y_{i}\text{log}P\left(Y=\text{1}|X_{i}\right)+\left(\text{1}-\;y_{i}\right)\text{log}\left(\text{1}-\;P\left(Y=\text{1}|X_{i}\right)\right)\right]\;\;\;\;\;\;\;\; \end{array}$$ (1.2)

### Validation and evaluation

To evaluate the models, the dataset was split into training (80%) and testing (20%) sets. Additionally, 10-fold cross-validation was applied to ensure more robust model performance. The following metrics were used for assessment:


$$\;\text{Accuracy}\;=\frac{TP\;+\;TN}{FP\;+\;FN\;+\;TP\;+\;TN}$$

$$\;\text{Precision}\;=\frac{TP}{TP+FP}$$

$$\;\text{Recall}\;=\frac{TP}{TP+FN}$$

$$\;\text{F1}\;\text{Score}\;=\text{2}.\frac{Precision*Recall}{Precision\;+\;Recall}$$

### Ethical approval

This study utilized publicly available data from the Bangladesh Demographic and Health Survey (BDHS), obtained from the DHS Program website (www.dhsprogram.com). The dataset is fully anonymized, and all identifying information was removed prior to analysis. The BDHS survey received ethical approval from the relevant national ethics committee in Bangladesh. Permission to access and use the dataset for independent research was granted by the DHS Program.

## Results and discussion

### Statistical overview

**Fig 3:**
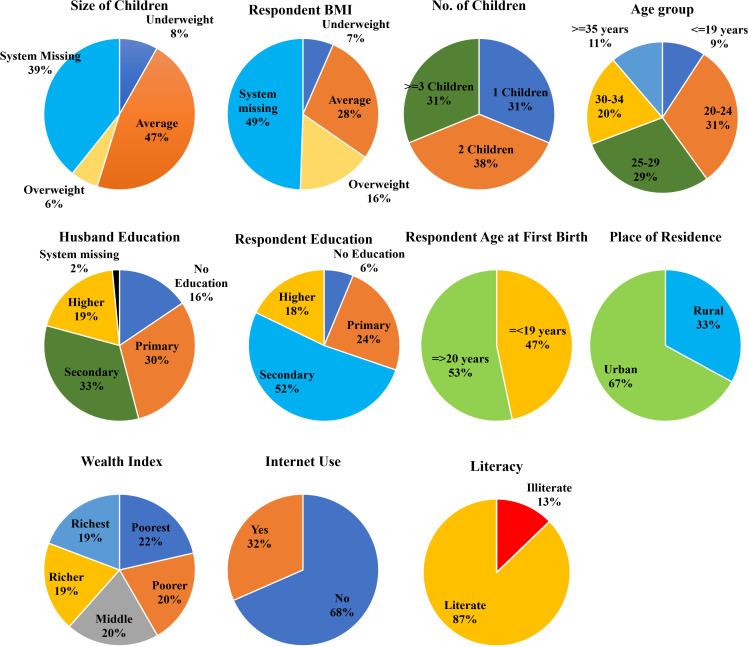
BDHS-2022: Descriptive Statistics of Socioeconomic and Health Determinants. The distribution of important socioeconomic and health variables from BDHS-2022 is shown in this image. These indicators include place of residence, wealth index, internet use, literacy, birth weight, BMI, number of children, age, education, and age at first birth.

The descriptive statistics of the total dataset (N = 8784) are summarized by their features as follows. This visualization in Fig 3 provides insights into various socioeconomic, demographic, and health-related variables, highlighting distributions among the respondents. It is evident from the charts that about 47% of the respondents had their first birth before 19 years of age, which is termed a teenage pregnancy. Moreover, 31% of the respondents have more than two children, 33% of the respondents are from rural areas, and 32% of the respondents reported using the internet during the 12 months preceding the interview.

### Correlations

**Fig 4:**
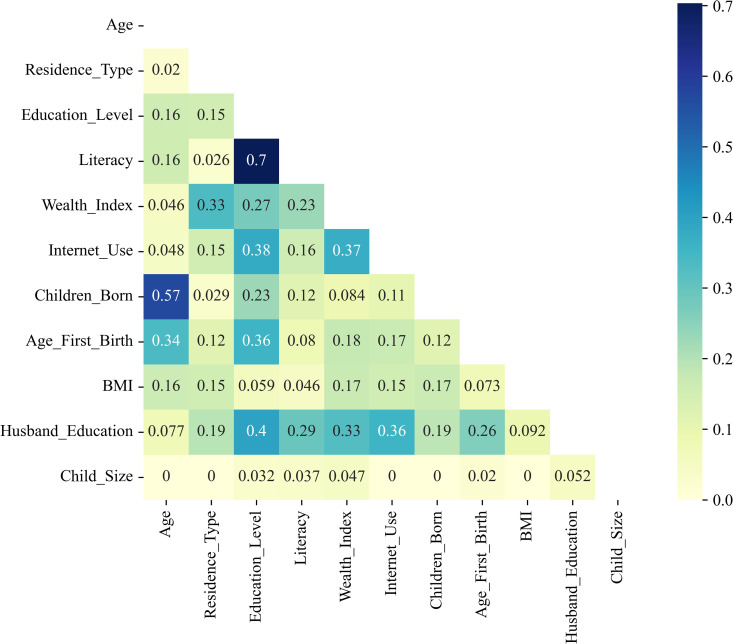
Cramér’s V correlation matrix showing the strength of association among categorical sociodemographic variables related to maternal and child characteristics in the BDHS-2022 dataset. Stronger associations are indicated by darker shades.

Cramér’s V was used to see how the categorical factors relate to one another and to the child’s size at birth. The values range from 0 to 1, with higher numbers showing stronger links. As shown in Fig 4, the clearest connections appear between literacy and education level (V = 0.70), age and number of children ever born (V = 0.57), and education level and husband’s education (V = 0.40), patterns that make social sense. In contrast, the child’s size shows only very weak ties with other variables (V < 0.06), suggesting that no single factor alone explains differences in birth size. Rather, it reflects the combined influence of many small, overlapping aspects of a mother’s social and economic life.

**Fig 5:**
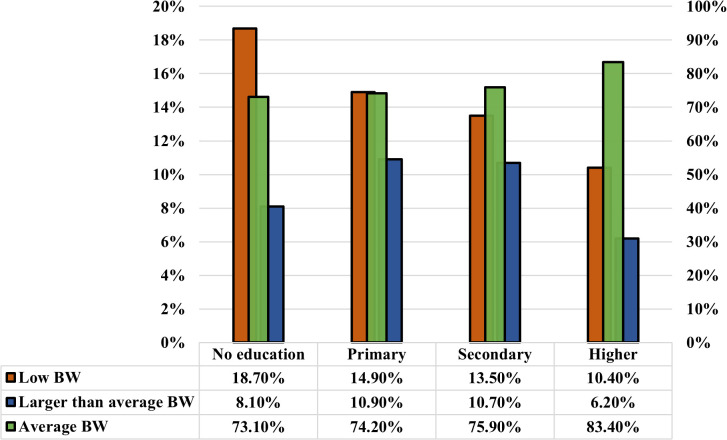
The correlation between low birth weight, average birth weight, and larger-than-average birth weight results and the respondent’s (Mother) education.

**Fig 6:**
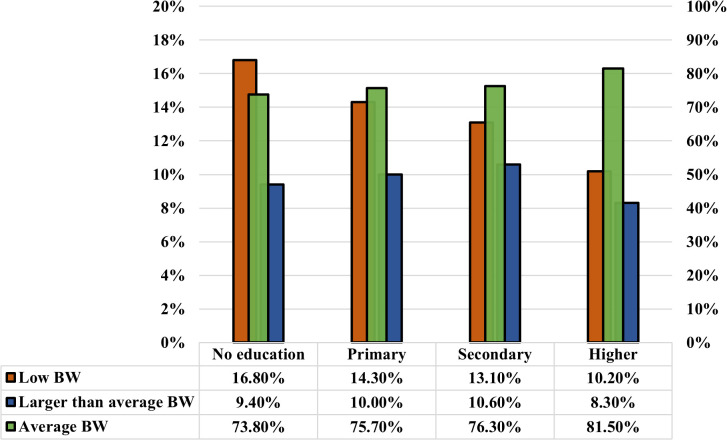
The correlation between low birth weight, average birth weight, and larger-than-average birth weight results and the respondent’s husband’s level of education.

**Fig 7:**
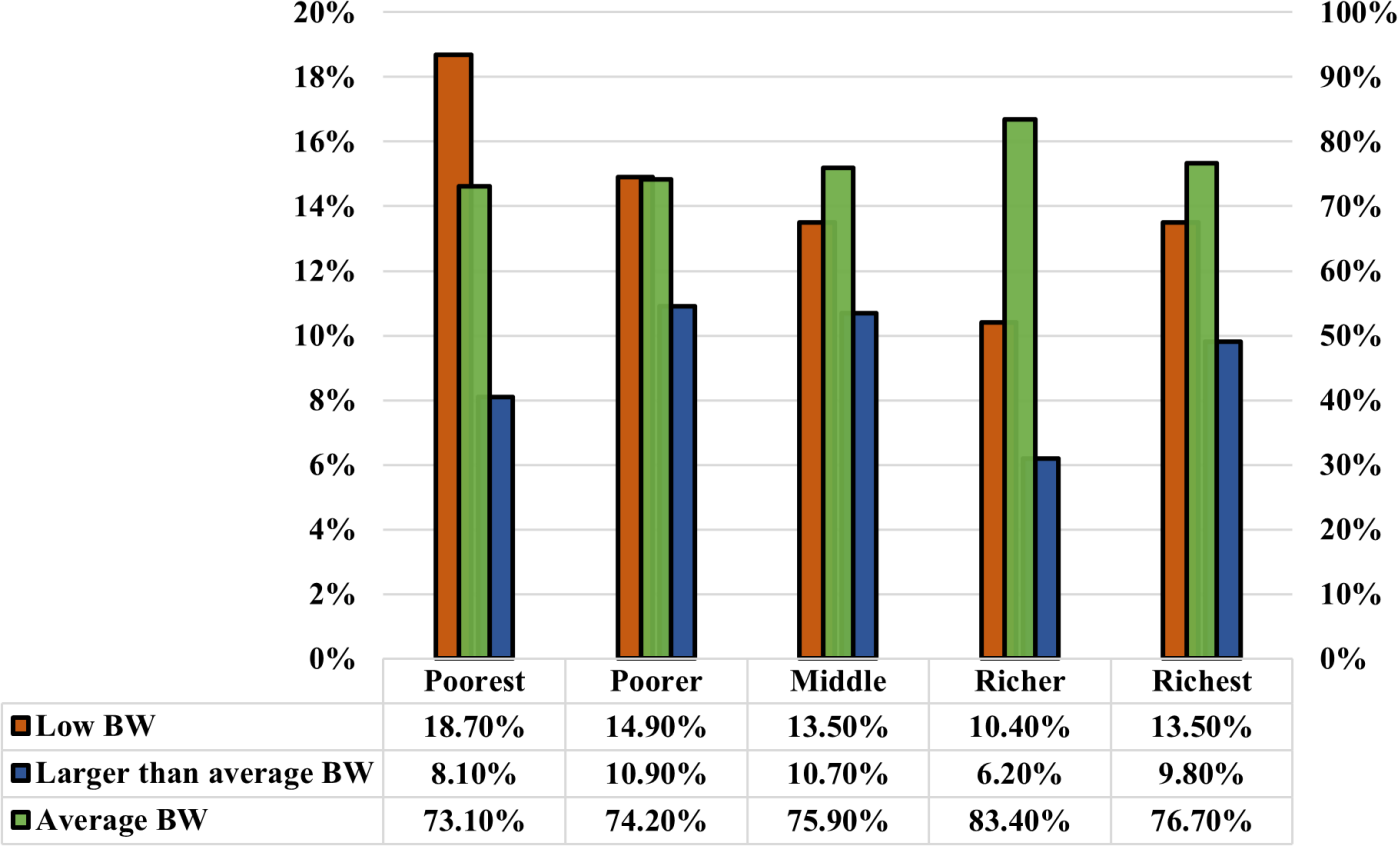
The correlation between the maternal wealth index and the outcomes of low birth weight, average birth weight, and larger-than-average birth weight.

**Fig 8:**
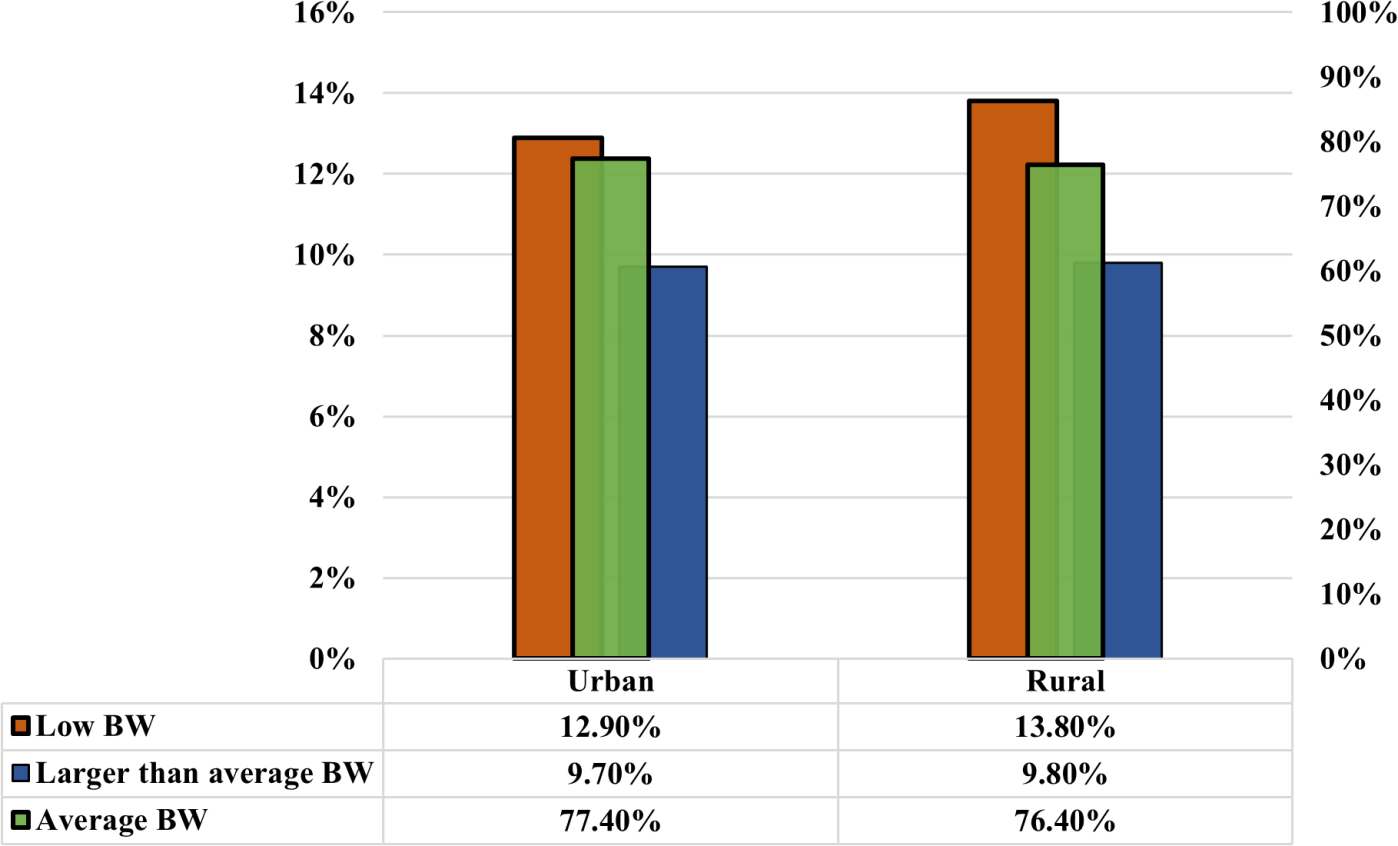
The correlation between the respondent’s place of residence and the outcomes of low birth weight, average birth weight, and larger-than-average birth weight.

As presented in Fig 5 and Fig 6, the analysis reveals a positive correlation between children’s birth weight and the education level of respondents and their husbands. Children’s size tends to improve as the respondent’s education level increases, as well as their husband’s education level. Notably, the prevalence of LBW is significantly lower (10.40%) among mothers with higher education compared to those with no education (18.70%). At the same time, the respondents whose husbands have no education have a similar trend in birth weight, with the highest proportion of LBW (16.80%), and fathers with higher education have significantly lower percentages of LBW (10.20%). Furthermore, the average birth weight per baby increases with higher education levels among parents. This study also found a significant and complex relationship between birth weight and wealth level. Birth weight is likely to increase with the rise in wealth level, up to the richest wealth quintile, while the poorest experience a decrease in birth weight. The prevalence of LBW decreases consistently with an increase in wealth. It is highest among the poorest group (18.70%) and lowest among the richer group (10.40%) (Fig 7). Moreover, the prevalence of low birth weight (LBW) is marginally higher in rural regions (13.8%) than in urban regions (12.9%) (Fig 8). The absence of notable differences in birth weight categories indicates that maternal health issues and access to healthcare are similar in urban and rural locations.

### District-wise spatial distribution of LBW in Bangladesh

**Fig 9:**
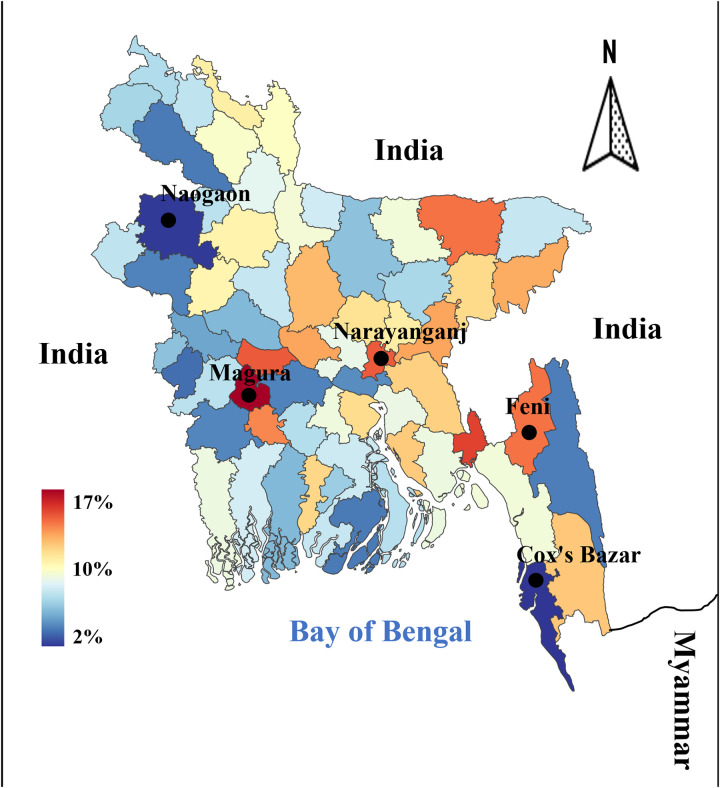
Bangladesh’s District-wise Spatial Distribution of Low Birth Weight (LBW) Density. Based on our dataset, this map shows the district-by-district density of low birth weight (LBW) cases in Bangladesh. LBW density is shown by the color gradient, where dark blue denotes a lower density (as low as 2%), and dark red denotes a higher density (up to 17%). Black dots indicate notable districts with noteworthy LBW densities, including Feni, Narayanganj, Magura, Cox’s Bazar, and Naogaon (BDHS-2022).

The spatial distribution of LBW across districts in Bangladesh, as presented in Fig 9, highlights significant geographic disparities. The density ranges from as low as 2% (dark blue) to as high as 17% (dark red), demonstrating that LBW is not uniformly distributed but concentrated in specific regions. Notably, the districts of Naogaon, Magura, Narayanganj, Feni, and Cox’s Bazar show distinct clustering of LBW prevalence. These districts, identified by black dots, represent hotspots of concern, aligning with findings from the Bangladesh Demographic and Health Survey (BDHS 2022).

Higher LBW prevalence is evident in certain central and southeastern districts (e.g., Narayanganj, Feni, and Cox’s Bazar). This pattern resonates with earlier studies indicating that urban-industrial regions (such as Narayanganj) may face elevated LBW risk due to air pollution, occupational exposures, and urban poverty [41,42]. Previously, it was found that the Chittagong division has the highest prevalence (16%) of LBW, whereas the Khulna division has the lowest prevalence of LBW (10.6%) [23]. For instance, Cox’s Bazar, a district with high LBW prevalence, has a substantial Rohingya refugee population, where limited maternal healthcare access and food insecurity exacerbate risks. Similarly, districts in the northern and southwestern regions (e.g., Naogaon, Magura) also exhibit elevated LBW densities, which may be linked to food insecurity, maternal malnutrition, and limited access to quality maternal health services, as highlighted in rural nutrition studies [23]. Magura and Naogaon, primarily agrarian districts, may reflect the role of low maternal BMI, limited dietary diversity, and early marriage, which are strongly correlated with LBW in rural Bangladesh [43].

### model training and prediction

This study employed two datasets to predict low birth weight (LBW): one containing only complete cases (N = 2,621) and another incorporating imputed values to enhance data coverage (N = 8,784). The predictive performance of multiple machine learning (ML) algorithms was evaluated using both the train–test split and a uniform repeated 10-fold cross-validation strategy, integrated with a comprehensive grid search procedure, which was applied consistently across all models to ensure fair and systematic hyperparameter optimization while effectively preventing data leakage between training and validation subsets. We compared performance metrics, including Accuracy, Precision, Recall, and F1-score, to assess potential overfitting across the training, testing, and cross-validation phases. The close alignment of these metrics across folds confirmed the models’ robust generalization capability and indicated that overfitting was minimal throughout the training process.

For the smaller dataset (N = 2,621), results from the train–test split revealed that Logistic Regression (LR), Support Vector Machine (SVM), Multilayer Perceptron (MLP), and AdaBoost each achieved the highest accuracy of 0.851429, with XGBoost demonstrating the strongest F1-score performance. Under 10-fold cross-validation, AdaBoost again outperformed the others, attaining an accuracy of 0.854635 and an F1-score of 0.789442, followed closely by LR, SVM, and MLP, which each achieved comparable accuracy (0.853492) and F1-scores (0.786029) (Table 2 and Table 3). When applied to the larger, imputed dataset (N = 8,784), all models exhibited substantially improved performance. Both evaluation methods indicated that LR, SVM, and MLP consistently achieved top-tier results, each reaching an accuracy of 0.920888 (train–test split) and 0.917919 (10-fold cross-validation), with corresponding F1-scores of 0.882677 and 0.882961. AdaBoost demonstrated slightly lower accuracy (0.920319) and F1-score (0.878635) under the train–test split, but comparable accuracy (0.917919) and a marginally higher F1-score (0.878855) during cross-validation (Tables 4 and 5).

**Table 2:** Several machine learning models’ performances were evaluated using Accuracy, Precision, Recall, F1 Score, R2 Score, and MSE using Train-Test Split on N = 2621 (excluding missing values).

Model	Accuracy	Precision	Recall	F1 Score	R2 Score	MSE
Logistic Regression	0.851429	0.724931	0.851429	0.783104		
Naive Bayes	0.817143	0.748022	0.817143	0.776454		
KNN	0.84	0.764094	0.84	0.786833		
Random Forest	0.822857	0.769122	0.822857	0.78909		
SVM	0.851429	0.724931	0.851429	0.783104		
Lasso Regression				-5.26E-05	0.126505	0.355675
Regression Tree				-0.7515	0.221561	0.470703
Neural Network (MLP)	0.851429	0.724931	0.851429	0.783104		
XGBoost	0.828571	0.773958	0.828571	0.79268		
AdaBoost	0.851429	0.800361	0.851429	0.786677		
Decision Tree	0.75619	0.775574	0.75619	0.765309		

**Table 3:** Using 10-Fold Cross-Validation on N = 2621 (excluding missing values), the performance of many machine learning models was evaluated by Accuracy, Precision, Recall, F1 Score, R2 Score, and MSE.

Model	Accuracy	Precision	Recall	F1 Score	R2 Score	MSE
Logistic Regression	0.853492	0.728451	0.853492	0.786029		
Naive Bayes	0.829063	0.759711	0.829063	0.78137		
KNN	0.841284	0.755138	0.841284	0.786733		
Random Forest	0.814202	0.747956	0.814202	0.776207		
SVM	0.853492	0.728451	0.853492	0.786029		
Lasso Regression				-0.00736	0.125229	0.352828
Regression Tree				-0.99067	0.24539	0.493824
Neural Network (MLP)	0.853492	0.728451	0.853492	0.786029		
XGBoost	0.819916	0.743339	0.819916	0.776451		
AdaBoost	0.854635	0.78061	0.854635	0.789442		
Decision Tree	0.724541	0.75317	0.724541	0.737882		

**Table 4:** Accuracy, precision, recall, F1 score, R2 score, and MSE were evaluated using Train-Test Split on N = 8784 (missing values were substituted with mode).

Model	Accuracy	Precision	Recall	F1 Score	R2 Score	MSE
Logistic Regression	0.920888	0.848034	0.920888	0.882961		
Naive Bayes	0.914058	0.847533	0.914058	0.87954		
KNN	0.91975	0.875207	0.91975	0.884529		
Random Forest	0.899829	0.862033	0.899829	0.878692		
SVM	0.920888	0.848034	0.920888	0.882961		
Lasso Regression				-0.00019	0.072867	0.269939
Regression Tree				-0.75865	0.128124	0.357944
Neural Network (MLP)	0.920888	0.848034	0.920888	0.882961		
XGBoost	0.916904	0.8754	0.916904	0.886834		
AdaBoost	0.920319	0.847993	0.920319	0.882677		
Decision Tree	0.85202	0.862909	0.85202	0.857348		

**Table 5:** The accuracy, precision, recall, F1 score, R2 score, and MSE of N = 8784 (missing values substituted with the mode) were evaluated using 10-Fold Cross-Validation.

Model	Accuracy	Precision	Recall	F1 Score	R2 Score	MSE
Logistic Regression	0.917919	0.842575	0.917919	0.878635		
Naive Bayes	0.914048	0.845136	0.914048	0.877091		
KNN	0.913935	0.845092	0.913935	0.876855		
Random Forest	0.900614	0.849543	0.900614	0.872672		
SVM	0.917919	0.842575	0.917919	0.878635		
Lasso Regression				-0.00398	0.075395	0.273609
Regression Tree				-0.82393	0.136085	0.36797
Neural Network (MLP)	0.917919	0.842575	0.917919	0.878635		
XGBoost	0.912909	0.851797	0.912909	0.877361		
AdaBoost	0.917919	0.850863	0.917919	0.878855		
Decision Tree	0.832196	0.852627	0.832196	0.842026		

In summary, AdaBoost and Logistic Regression demonstrated the highest predictive accuracy and stability across datasets and validation techniques. Specifically, AdaBoost achieved up to 85.46% accuracy and an F1-score of 0.79 with the smaller dataset, while Logistic Regression reached approximately 91.08% accuracy and an F1-score of 0.88 on the larger, imputed dataset. Logistic Regression proved advantageous for its interpretability and consistent performance with extensive data, whereas AdaBoost exhibited superior adaptability and balance between precision and recall. Together, these models provided the most dependable and practical framework for accurately identifying mothers at risk of delivering low-birth-weight infants.

### Feature importance analysis across machine learning models

**Fig 10:**
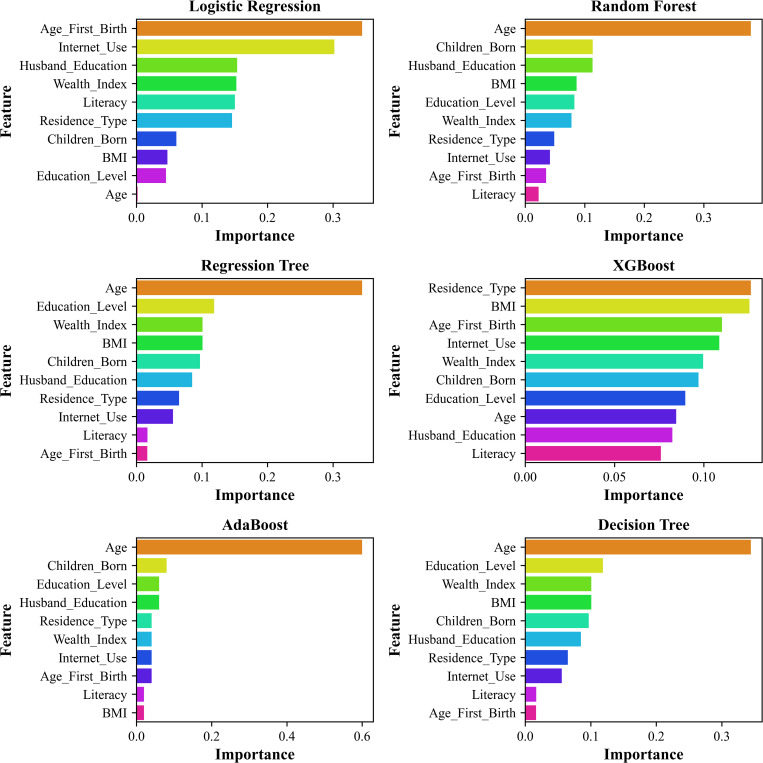
Highlighting the significance of each feature to model predictions by comparing feature importance across various machine learning models with N = 2621.

**Fig 11:**
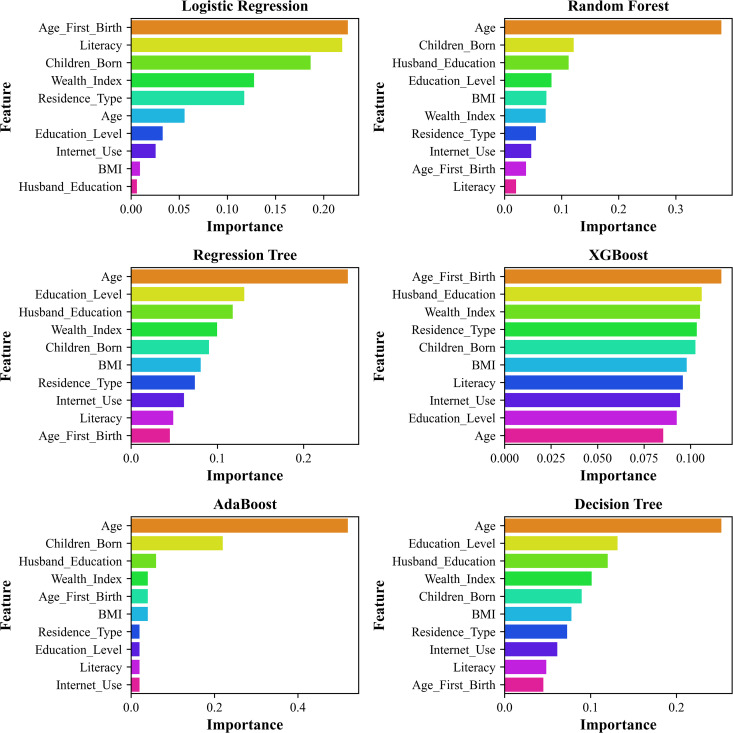
The significance of each attribute in relation to model predictions is highlighted for N = 8784 across several machine learning models.

The rankings of feature relevance for datasets based on six machine learning models, like LR, RF, RT, XGBoost, AdaBoost, and DT, are presented in Fig 10 and Fig 11. These models were used to assess the relative influence of various sociodemographic and economic factors on infant birth weight. In LR Fig 10, Age at First Birth, Internet Use, and Husband’s Education emerged as the most significant predictors. Similarly, in Fig 11, Age at First Birth, Literacy rate, and the number of children ever born were identified as the most influential. This variation suggests that feature importance is sensitive to differences in data distribution. Tree-based models, such as RF and DT, consistently identified the age of the respondent and the number of children ever born as the most critical variables for RF, and the age of the respondent and the education level of the husband as the most critical variables for DT, in both datasets. These results indicate that tree-based models prioritize demographic factors when making predictions. Similarly, AdaBoost models highlighted the age of respondents and the number of children ever born as significant determinants for two datasets, emphasizing their role in the studied phenomenon. XGBoost, known for its advanced gradient-boosting mechanism, presented a more dispersed ranking of feature importance for two datasets, where it revealed the resident type of respondents, followed by BMI, as the highly influential variables for the smaller dataset (N = 2621), and age at first birth, followed by husband education, for the other dataset (N = 8784). RT ranked the age of the respondent and the education level of the husband as the highest influential features for both datasets. In Fig 10 and Fig 11, except for XGBoost, the other models show consistency in selecting key determinants responsible for LBW in both datasets, where only LG found age at first birth, and the others found Age of the respondent. These findings suggest that boosting techniques can capture complex feature interactions beyond the scope of traditional decision trees and regression models.

The variation in feature importance rankings observed across models such as Logistic Regression, XGBoost, and AdaBoost can be attributed to inherent differences in their underlying learning mechanisms and how they capture relationships among predictors. Specifically, Logistic Regression, being a linear model, estimates the independent and additive effects of each variable on the outcome, assuming linearity and minimal interaction among features. In contrast, tree-based and boosting algorithms (e.g., Random Forest, XGBoost, and AdaBoost) can model nonlinear associations and complex feature interactions through recursive partitioning and hierarchical splitting, naturally leading to variation in feature rankings. These differences do not indicate inconsistency but highlight model-specific sensitivities to data structure and feature interdependence. Notably, despite the methodological divergence, all models consistently identified key sociodemographic determinants, particularly maternal age, age at first birth, and husband’s education, as the most influential predictors of low birth weight (LBW), reaffirming their robustness and central role in maternal and child health outcomes.

**Fig 12:**
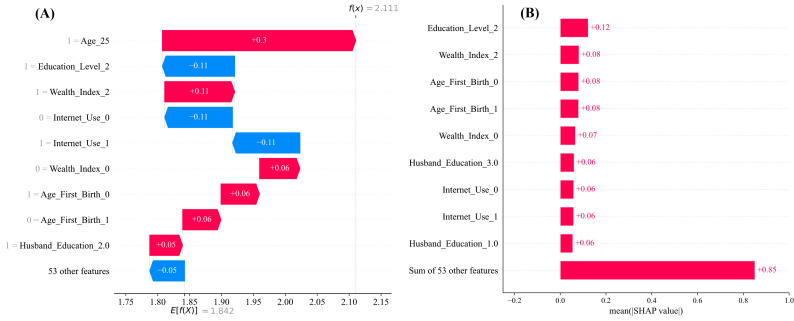
SHAP feature importance and summary plots of the top 10 features from the Logistic Regression model. (A) SHAP waterfall plot showing the contribution of individual features for a single prediction instance. (B) SHAP bar plot summarizing the mean absolute SHAP values of the top 10 features across the dataset. The features are listed in order of importance. The SHAP value of a feature represents the extent to which it contributes to the prediction. A positive SHAP value indicates an increased likelihood of predicting low birth weight (LBW), whereas a negative SHAP value indicates a decreased likelihood.

To reduce the ambiguity of feature importance, this study adopted SHAP (Shapley Additive exPlanations) analysis for the Logistic Regression model found in this study to classify LBW outcomes, and the output is presented in Fig 12. Panel A waterfall plot illustrates the SHAP (SHapley Additive exPlanations) values that detail the contribution of each feature to the model’s prediction for a specific individual, thereby explaining why the model predicted low birth weight (LBW) in this case. The base value, E [f(x)] = 1.842, represents the average model output in log odds across all individuals in the dataset. The predicted value for this instance is f(x) = 2.111, which suggests an elevated risk of LBW. Features that increase the prediction’s likelihood of LBW are highlighted in red and represent positive SHAP values. In contrast, features shown in blue have negative SHAP values and contribute to a decreased likelihood of LBW. Among all features, the individual’s age group, specifically 25 years old (Age_25 = 1), has the most substantial positive contribution, increasing the prediction by +0.30 log-odds. In contrast, some features act as protective factors, reducing the risk of LBW. Notably, having secondary or higher education (Education Level 2) and access to the internet contribute negative SHAP values, pulling the prediction downward and indicating a lower probability of LBW. Additional features, such as the Wealth Index, Age at First Birth, and Husband’s Education Level, also affect the prediction, though their contributions are relatively minor. Overall, while being 25 years old increases the predicted risk of LBW the most, the presence of favorable socio-demographic conditions like higher maternal education and internet access partially offsets this risk.

Bar Plot (Fig 12 Panel B) presents the mean absolute SHAP values for the top 10 features influencing the logistic regression model’s predictions across the entire dataset. It reveals which variables play the most critical role in determining the likelihood of low birth weight (LBW). Among these, maternal education, specifically Education Level 2 (secondary or higher education) emerges as the most influential feature, with a mean SHAP value of 0.12. This indicates that maternal education consistently has a strong impact on the model’s predictions across many individuals. Other key variables include Wealth_Index_2 (representing middle-class families), Age_First_Birth_0 (representing teenage mothers), Internet_Use_1 (indicating that the mother uses the internet), and Husband_Education_1 (Indicating a Husband with Primary Education). Collectively, the remaining 53 features contribute a combined SHAP value of 0.85, suggesting that while the model draws on a broad array of inputs, only a handful of predictors exert dominant influence. The analysis underscores the pivotal role of maternal education in reducing the risk of LBW, followed by household economic status and the age at which mothers give birth for the first time. These findings underscore the significance of socio-demographic factors in influencing birth outcomes and can inform targeted public health interventions designed to enhance maternal and child health in Bangladesh.

These findings align with previous research, indicating that young maternal age (<19 years) is a significant risk factor for LBW [44]. These results are consistent with a study conducted in Southeast Asia, which found that adult women from wealthier families, with well-educated partners, and living in urban areas had a lower likelihood of delivering LBW infants [45,46]. The high prevalence of LBW among socioeconomically disadvantaged families may be attributed to poor living conditions and inadequate maternal health care. Teenage mothers, belonging to the poorest wealth groups, residing in rural areas where they lacked access to modern amenities such as the internet, were more likely to give birth to LBW infants. Furthermore, internet use emerged as a significant factor influencing birth outcomes. Digital technology facilitates access to health information and guidance on prenatal care, which can improve maternal health behaviors [47]. Mothers who use the internet are more likely to access health information, antenatal care, danger signs, and safe pregnancy practices. Nowadays, various online forums and social media groups enable mothers to learn from peer experiences and take action earlier if complications arise. However, excessive internet use has been linked to negative health effects, potentially impacting birth weight outcomes [48]. In addition, grand multiparous mothers were found to have a higher likelihood of delivering LBW infants compared to multiparous women, whereas primiparous and nulliparous women had a lower risk [49,50]. Additionally, maternal BMI was found to be a significant predictor of birth weight. Underweight mothers, who often experience nutritional deficiencies, are at an increased risk of delivering LBW infants, whereas overweight mothers are more susceptible to pregnancy complications and non-communicable diseases, which may also contribute to LBW outcomes [51]. Research has also shown that the prevalence of macrosomia (large-size infants) is higher among overweight and obese mothers [52].

The observed variations in feature importance rankings across different models highlight the critical role of model selection in predictive analytics. Logistic Regression places greater emphasis on behavioral and socioeconomic factors, whereas tree-based ensemble methods prioritize demographic variables. These findings highlight the importance of striking a balance between interpretability and predictive accuracy when selecting an optimal model for public health interventions and policymaking.

### Policy suggestions

This study identifies key strategies to reduce low birth weight (LBW) in Bangladesh. Targeted maternal health programs should focus on hotspot districts, such as Magura, Narayanganj, Feni, and Cox’s Bazar, with an emphasis on improved nutrition, prenatal care, and equitable resource allocation. Community-based education should promote maternal and paternal literacy, awareness of adolescent pregnancy risks, and delayed childbearing. Economic and nutritional support for low-income mothers through financial empowerment and regular nutritional monitoring—can further enhance birth outcomes.

Using an ML model to identify possible risky mothers who are going to deliver a LBW baby would introduce a milestone in improving reproductive health in Bangladesh. It would be easier to bring them under comprehensive intervention with improved healthcare infrastructure, expanded telemedicine, and trained community health workers to provide culturally appropriate counseling, which is essential for both rural and urban service delivery. Strengthening adolescent health programs that prioritize family planning and reproductive education can directly reduce adolescent pregnancies, a major LBW predictor. District-specific, place-based interventions, including improved nutrition in food-insecure areas and targeted health services for marginalized groups, can substantially lower LBW prevalence and advance national maternal and child health goals.

### Limitation

This study acknowledges certain limitations that could be addressed in future research. The inclusion of additional maternal health indicators, such as anemia, diabetes, hypertension, gestational period, and other medical conditions, could enhance the predictive accuracy of the models. Furthermore, incorporating factors related to pregnancy intention, maternal knowledge of pregnancy management, and access to healthcare services would provide a more comprehensive understanding of the determinants of LBW. Another limitation is the reliance on available sociodemographic variables, which may not fully capture the complex interplay of biological and behavioral factors influencing birth outcomes. Only 14% of infants were LBW, indicating moderate class imbalance. No resampling or class-weight adjustment was applied; therefore, although overall accuracy was high, precision and recall for the LBW class may be underestimated. A potential limitation of this study lies in the exclusive use of SHAP for model interpretability [53], without incorporating alternative explanation techniques such as LIME. While SHAP offers a theoretically grounded and globally consistent framework for quantifying each feature’s contribution to model predictions, the absence of comparative analyses with other interpretability approaches like LIME’s locally perturbed explanations may limit the breadth of interpretive perspectives on how input variables influence the predicted likelihood of low birth weight. Future studies should consider SMOTE or class-weighting to improve sensitivity for rare outcomes

## Conclusions

This study utilized the Bangladesh Demographic and Health Survey (BDHS) 2022 dataset to develop predictive machine-learning models for assessing the likelihood of LBW based on maternal sociodemographic characteristics. It is found that the dataset excluding missing values produces cleaner, stronger models than those with mode imputation. The 10-fold cross-validation method was found to be a more stable and reliable estimate of performance compared to the train-test split, which may be due to its averaging over 10 different splits. Among the diverse machine learning algorithms employed in this study, AdaBoost emerged as the most robust and consistently reliable model, exhibiting superior predictive performance in identifying mothers at heightened risk of delivering low birth weight (LBW) infants. Its ensemble-based architecture, which adaptively combines multiple weak learners to minimize classification errors, enabled AdaBoost to effectively capture complex, nonlinear relationships among maternal, socioeconomic, and healthcare-related factors. This stability and adaptability underscore AdaBoost’s potential as a powerful decision-support tool for early detection and intervention in maternal and neonatal health. Key maternal factors identified as significant predictors of LBW include age, age at first birth, level of education, wealth index, body mass index (BMI), and parity. These findings align with previous research highlighting the complex interplay of socioeconomic, behavioral, and demographic factors in determining birth weight outcomes.

The study further emphasizes the role of predictive analytics in public health interventions. By leveraging machine learning models, policymakers and healthcare professionals can proactively identify high-risk mothers throughout the country and implement targeted interventions to reduce LBW prevalence. To increase the age at first birth, measures such as improving mothers’ education, enhancing wealth levels, strengthening maternal and child health programs, deploying community health workers to monitor vulnerable mothers, and providing financial support for antenatal care can be effective in addressing this issue. This study can also guide future researchers in creating more robust models by incorporating additional health variables, such as anemia, hypertension, and gestational period.
